# Preferences for methadone clinics among drug users in Vietnam: a comparison between public and private models

**DOI:** 10.1186/s12954-019-0355-x

**Published:** 2020-01-06

**Authors:** Tuan Anh Le, Tuan Anh Nguyen, Anh Duc Dang, Cuong Tat Nguyen, Hai Thanh Phan, Giang Thu Vu, Trang Huyen Thi Nguyen, Carl A. Latkin, Cyrus S. H. Ho, Roger C. M. Ho, Bach Xuan Tran, Jiangbo Ying, Melvyn W. B. Zhang

**Affiliations:** 1 National Institute of Hygiene and Epidemiology, Hanoi, Vietnam; 2 Institute for Preventive Medicine and Public Health, Hanoi Medical University, Hanoi, Vietnam; 3 Institute for Global Health Innovations, Duy Tan University, Da Nang, Vietnam; 4 Center of Excellence in Evidence-based Medicine, Nguyen Tat Thanh University, Ho Chi Minh City, Vietnam; 5 Center of Excellence in Pharmacoeconomics and Management, Nguyen Tat Thanh University, Ho Chi Minh City, Vietnam; 6 Bloomberg School of Public Health, Johns Hopkins University, Baltimore, MD USA; 7 Department of Psychological Medicine, National University Hospital, Singapore, Singapore; 8 Center of Excellence in Behavioral Medicine, Nguyen Tat Thanh University, Ho Chi Minh City, Vietnam; 9 Department of Psychological Medicine, Yong Loo Lin School of Medicine, National University of Singapore, Singapore, Singapore; 10 Institute for Health Innovation and Technology (iHealthtech), National University of Singapore, Singapore, Singapore; 11 National Psychiatry Residency Program, National Healthcare Group, Singapore, Singapore; 12 Family Medicine and Primary Care, Lee Kong Chian School of Medicine, Nanyang Technological University, Singapore, Singapore

**Keywords:** Methadone, MMT, Preference, Vietnam

## Abstract

**Background:**

Methadone maintenance treatment (MMT) has been proven to be effective in treating opioid dependence. In Vietnam, MMT services are provided primarily by public clinics, with only one private MMT clinic established in recent years. Assessing the preferences of patients for different MMT models is important in evaluating the feasibility of these models. This study measured the preferences of drug users enrolling in public and private MMT clinics in Vietnam and examines the related factors of these preferences.

**Methods:**

A cross-sectional study was performed on 395 participants at 3 methadone clinics in Nam Dinh. Data about the preferences for MMT models and sociodemographic characteristics of participants were collected. Exploratory factor analysis was employed to explore the construct validity of the questionnaire. The chi-square test and Mann-Whitney test were used for analyzing demographic characteristics and preferences of participants. Multivariate logistic regression identified factors associated with participants’ preferences.

**Results:**

Half the participants received MMT treatment in a private facility (49.4%). Two preference dimensions were defined as “Availability and convenience of service” and “Competencies of clinic and health professionals”. Self-employed patients were more likely to consider these two dimensions when choosing MMT models. Only 9.9% of participants chose “Privacy” as one of the evaluation criteria for an MMT facility. Compared to public clinics, a statistically higher percentage of patients in the private clinic chose the attitudes of health workers as the reason for using MMT service (34.7% and 7.6% respectively). Mean score of satisfaction towards MMT services was 8.6 (SD = 1.0), and this score was statistically higher in a public facility, compared to the private facility (8.7 and 8.4 respectively).

**Conclusions:**

The study highlighted patterns of patient preferences towards MMT clinics. Compared to the public MMT model, the private MMT model may need to enhance their services to improve patient satisfaction.

## Background

Methadone maintenance treatment (MMT) is an essential and cost-effective therapy for patients with opioid dependence [1, 2]. Methadone is considered by the World Health Organization (WHO) as a priority drug for the management of opioid dependence [3]. Methadone, being a full opioid agonist, can help individuals transition over from illicit opioids and prevents withdrawal symptoms. Vietnam is one of the countries with the highest rate of human immunodeficiency virus (HIV) transmission through injecting drug users, and the HIV prevalence among people with inject drugs (PWID) has been estimated to be around 20% [4]. MMT programs in Vietnam have demonstrably not only reduced opiate use and decreased risky sexual behaviors but also help improve patients’ quality of life and overall social stability [4–7]. Since the introduction of MMT in Vietnam in 2008, over 51,000 patients have received MMT in 280 nationwide MMT clinics [8]. With strong political will and commitment, the Vietnamese government has plans to scale-up the coverage of MMT program with a target of 80,000 drug users [9]. The aforementioned plan might not be feasible given there being a significant reduction in foreign aids in the next few years [10]. To address this challenge, there need to be well-thought-out strategies to optimize MMT service models, with a reduction in the operational resources, to ensure the sustainability of the existing MMT program.

In Vietnam, MMT services have been provided by public clinics, with the establishment of only one private MMT clinic in Nam Dinh province in recent years. Public and private models in Vietnam can differ in several aspects, including privacy, convenient opening hours, equipment, health worker skills and attitudes, and extent of financial support. Both public and private models have their strengths. The private model may allow suitable patients more immediate access to treatment [11]. However, the mean cost of the private model is likely higher than that of the public model [12]. To assess the performance of health care services, information about patient characteristics, experience, and satisfaction are essential [13]. Patient preference and satisfaction can assist policymakers in understanding patients’ needs and identifying gaps to improve the quality of health care service [14, 15]. It could also help healthcare providers in predicting the retention, adherence, and treatment outcome [14, 16].

Assessing the preferences of patients for different MMT service models is crucial in evaluating the feasibility of implementing these models. This is especially of importance in the context of Vietnam, as there has been controversy around the establishment of private MMT clinic, mostly on how poor and unemployed illicit drug users will be able to afford the treatment that has long been provided for free by the government. However, to our knowledge, there has been no prior literature published on patients’ preferences of public and private models in Vietnam. Thus, this study aimed to examine factors related to these preferences of drug users enrolling in MMT programs for public and private models and examine the related factors of these preferences.

## Methods

### Study setting and subjects

A cross-sectional study was performed from January to September 2018 in Nam Dinh province, one of the largest epicenters providing human immunodeficiency virus and HIV/AIDS surveillance and treatment services in the North of Vietnam. The study was conducted at three methadone clinics including Giao Thuy district health center (public model), Giao Thuy district center for social evils prevention (public model), and Dai Dong private health facility (private model). The eligibility criteria for selecting outpatient clinic sites were (1) being able to afford methadone treatment following the official guidelines of the Vietnamese Ministry of Health and (2) the patient had been on methadone treatment for at least 12 months.

We used convenience sampling to recruit participants who met the following eligibility criteria: (1) being 18 years old or above, (2) receiving methadone treatment from those clinics mentioned above, (3) agreeing to participate in the study, and (4) ability to adequately communicate with the data collector. The exclusion criteria included those who suffered from a serious illness. A total of 395 participants agreed to join the study. The percentage of patients in each facility was 49.4% (Dai Dong private health facility), 25.3% (Giao Thuy district health center), and 25.3% (Giao Thuy district center for social evils prevention).

### Measure and instruments

Participants were invited to participate in 20-min face-to-face interviews. The data collectors were researchers who underwent extensive training. We did not invite local methadone service providers to participate in data collection to avoid social desirability bias. We approached participants when they visited clinics for medication or to receive counseling. We identified eligible criteria for selecting participants for the study based on the health staff’s feedbacks. These participants were invited into a small counseling room in order to protect their confidentiality. After the interviewer explained the purpose of the study, that of the benefits, and drawbacks from participating, participants were asked to join the study. Participants provided verbal informed consent. To ensure participants’ confidentiality, the consent process took place in a comfortable room with restricted access, which allowed participants to have privacy when deciding whether to join the project.

We conducted a pilot survey among 20 participants of different ages, genders, and occupations. Minor changes were made to some of the wordings, so that it was appropriate given participants’ preferences and culture. The questionnaire included the following information:

(i) Socio-economic characteristics

Participants self-reported their age, gender, education, marital status, occupation, and monthly income.

(ii) Network of methadone maintenance treatment facilities

In order to examine participants’ selection for choosing MMT health facility, we asked them a series of questions. Each answer ranged from 1 “very important” to 5 “very unimportant”. These items were then used for exploratory factor analysis (EFA). The general evaluation (determined by EFA) score for each domain was calculated as the mean score of the component questions. Then, in each domain, we summed all items before dividing to the number of items to calculate the score of this domain. The range score of each domain was from 0 to 10.

(iii) Participants’ preferences for other methadone maintenance treatment facilities

In order to investigate the preference for other MMT health facilities, participants were asked about their intentions to switch to another MMT facility, which MMT facility that they wanted to switch from, and the main reason for such change. We also asked participants whether they moved from another MMT health facility to their current facility and the most crucial reason for such movement.

### Statistical analysis

Data were analyzed by STATA version 12 (Stata Corp. LP, College Station, USA). In this study, we employed EFA to explore the construct validity of the questionnaire. Principal component analysis was used to extract those factors using a threshold of an eigenvalue of 1.2, where the curve was flattened. The threshold was defined by the screen test. We used an Orthogonal Varimax rotation with Kaisers’ normalization to re-organize items into a scale to increase the interpretability of our results. A value of 0.5 was used as the cut-off point for factor loadings. Additionally, a cross-loading for one item was conducted and assigned to the proper domain regarding its nature and the overarching dimension. Internal consistency of the instrument was measured by using Cronbach’s alpha.

A chi-square test, a Fisher exact test, and a Mann-Whitney test were used for analyzing demographic characteristics of participants as well as participants’ preferences for other MMT facilities. We also applied multivariate logistic regression to identify factors associated with participants’ preferences for other MMT facilities. We applied a forward stepwise selection strategy to remove non-significant factors, the *p* value of the log-likelihood ratio test was set as less than 0.2, and this was the threshold to include a variable. A *p* value < 0.05 was considered as statistical significance.

## Results

Table 1 describes the information about demographic and substance use characteristics of participants in this study. The percentage of participants receiving MMT treatment in the private facility was similar to that of those in the public facilities (49.4% and 50.6%). The majority of participants had secondary education (60%) and lived with their spouse/partners (77.0%). About one third of respondents were self-employed (35.2%), followed by blue collar/farmer (23.3%). The proportion of five groups of quintile monthly income was similar, approximately 20%. Nearly two thirds of MMT patients had a history of injecting drugs (63.8%), and only 6% of patients still used drugs. More than 80% and half of the respondents smoked and drank alcohol. Median (IQR) of age and MMT duration were 39 (33–46) and 3 (1–5).

**Table 1:** Demographic and substance abuse characteristics of participants

Characteristics	Private facility		Public facility		Total		*p* value
	*n*	%	*n*	%	*n*	%	
Total	195	49.4	200	50.6	395	100.0	
Education							
Under secondary school	33	16.9	33	16.5	66	16.7	0.94*
Secondary school	118	60.5	119	59.5	237	60.0	
Above secondary school	44	22.6	48	24.0	92	23.3	
Marital status							
Single	38	19.5	29	14.5	67	17.0	0.07*
Live with partners/spouse	150	76.9	154	77.0	304	77.0	
Divorced/widow	7	3.6	17	8.5	24	6.1	
Occupation							
Unemployment	13	6.7	20	10.0	33	8.4	0.06*
Self-employed	63	32.3	76	38.0	139	35.2	
Blue collar worker/farmer	45	23.1	47	23.5	92	23.3	
Business	11	5.6	17	8.5	28	7.1	
Others	63	32.3	40	20.0	103	26.1	
Family financial condition							
Wealthy	8	4.1	3	1.5	11	2.8	0.10*
Normal	152	78.0	148	74.0	300	76.0	
Poor	35	18.0	49	24.5	84	21.3	
Quintile monthly family income							
Poorest	39	20.0	41.0	20.5	80	20.3	0.89*
Poor	37	19.0	45.0	22.5	82	20.8	
Middle	42	21.5	39.0	19.5	81	20.5	
Rich	43	22.1	39.0	19.5	82	20.8	
Richest	34	17.4	36.0	18.0	70	17.7	
Ever injected drugs	121	62.1	131	65.5	252	63.8	0.48*
Alcohol drink	114	58.5	97	48.5	211	53.4	0.05*
Smoke	163	83.6	157	78.5	320	81.0	0.20*
Concurrent drug use	15	7.7	8	4.0	23	5.8	0.12*
	Median	IQR	Median	IQR	Median	IQR	*p* value
Age	38	33–44	41	34–48	39	33–46	0.01^#^
Monthly family income (USD)	344	215–430	301	215–430	344	215–430	0.50^*#*^
Age at onset of drug use	25	20–30	25	21–31	25	20–31	0.05^*#*^
MMT duration (years)	2	1–5	3	2–6	3	1–5	0.02^*#*^

*Chi-square test, ^#^Mann-Whitney rank sum test

Table 2 illustrates the evaluation criteria of MMT patients about their MMT facility. According to factor analysis, there were two dimensions, namely “Availability and convenience of service” and “Competencies of clinic and health professionals.” Cronbach’s alpha of two domains were 0.81 and 0.8, respectively, and the mean scores (SD) were 6.43 (1.38) and 7.09 (1.38). Among participants, 82% selected “Able to present comprehensive care,” followed by “Convenient opening hours” (81.8%) and “Able to treat other diseases” (77.0%). Only 9.9% of participants chose “Privacy.”

**Table 2:** MMT facility evaluation criteria among patient

Characteristics	*n*	%	Availability and convenience of service	Competencies of clinic and health professionals
Evaluation items				
Privacy	39	9.9	0.59	
Convenient opening hours	323	81.8		0.63
Health worker skills and abilities	233	59.0		0.91
Health worker attitude	196	49.6		0.89
Facilities, equipment	203	51.4	0.62	
Able to treat other diseases	304	77.0	0.84	
Able to present comprehensive care	324	82.0	0.86	
Service to support to adhere to treatment	186	47.1	0.87	
Support to financial/procedures	211	53.4	0.89	
Reliability				
Cronbach’s alpha			0.81	0.80
Domain score				
Mean			6.43	7.09
SD			1.38	1.38

The information about MMT facility preference among MMT patients is provided in Table 3. Only 1.3% of the participants planned to change MMT facility. In total, 39% of the patients had received MMT treatment in other MMT facility in the past, and majority were treated in an MMT facility within the district (94.2%). Shorter distance was considered as the important reason to use service in their current MMT facility (94.2%), followed by convenient hours (22.7%) and attitude of health worker (20.8%). The percentage of those who chose attitudes of health workers as the reason for using MMT service in a public facility was statistically higher than those in a private facility (34.7% and 7.6% respectively). The mean score of satisfaction towards MMT services was 8.6 (SD = 1.0), and this score was statistically higher in the public facilities, compared to the private facility (8.7 and 8.4 respectively).

**Table 3:** MMT preference among patient

Characteristics	Private facility		Public facility		Total		*p* value
	*n*	%	*n*	%	*n*	%	
Know other MMT facility in Nam Dinh	155	79.5	172	86.0	327	82.8	0.09*
Plan to change MMT facility	0	0.0	5	2.5	5	1.3	0.06^¢^
Had received MMT treatment at other MMT facility	79	40.5	75	37.5	154	39.0	0.54*
Location of last MMT facility							
In the same district	78	98.7	67	89.3	145	94.2	0.01^¢^
In other districts within the province	0	0.0	7	9.3	7	4.6	
In other provinces	1	1.3	1	1.3	2	1.3	
Reason to use service in current MMT facility							
Near distance	78	98.7	67	89.3	145	94.2	0.02^¢^
Convenient opening hours	13	16.5	22	29.3	35	22.7	0.06*
Health worker skills and abilities	7	8.9	15	20.0	22	14.3	0.05*
Health worker attitude	6	7.6	26	34.7	32	20.8	< 0.01*
Facilities, equipment	1	1.3	8	10.7	9	5.8	0.02*
Able to treat other diseases	1	1.3	1	1.3	2	1.3	1.00^¢^
Able to present inter-professional care	0	0.0	1	1.3	1	0.7	0.49^¢^
Most important reason to change to current MMT facility (*n* = 154)							
Near distance	76	96.2	54	72.0	130	84.4	< 0.01^¢^
Health worker capacity and attitude	2	2.5	12	16.0	14	9.1	
Others	1	1.3	9	12.0	10	6.5	
	Mean	SD	Mean	SD	Mean	SD	*p* value
MMT service satisfaction	8.4	1.0	8.7	0.9	8.6	1.0	< 0.01^#^

*Chi-square test, ^¢^Fisher exact test, ^#^Mann-Whitney rank sum test

Table 4 presents factors that associated with the preference for choosing an MMT facility among MMT patients. Participants who were self-employed were more likely to choose “Availability and convenience of service” (Coef. = 0.5, 95%CI = 0.2, 0.8) and “Competencies of clinic and health professionals” (Coef. = 0.59, 95% CI = 0.29, 0.89). A similar pattern was found for people who drank alcohol. Those who worked other jobs, had injected drugs, and smoked were less likely to choose the two these two criteria of preference. People who were working other jobs, being in a poor income group, and had a higher level of satisfaction with MMT service had a lower likelihood of choosing “Availability and convenience of service.”

**Table 4:** Factors associated with preference for MMT facility among patients

Characteristics	Availability and convenience of service		Competencies of clinic and health professionals	
	Coef.	95% CI	Coef.	95% CI
Marital status (vs single)				
Live with partners/spouse	− 0.25*	− 0.55; 0.04		
Divorced/widow			0.53**	0.01; 1.04
Occupation (vs unemployment)				
Self-employed	0.50***	0.20; 0.80	0.59***	0.29; 0.89
Business	− 0.51*	− 1.02; 0.00	− 0.50*	− 1.01; 0.01
Other	− 0.75***	− 1.08; − 0.42	− 0.88***	− 1.21; − 0.55
Quintile monthly family income (vs poorest)				
Poor	− 0.33**	− 0.63; − 0.03		
Ever injected drugs (yes vs no)	− 0.29**	− 0.55; − 0.03	− 0.38***	− 0.63; − 0.12
Alcohol drink (yes vs no)	0.39***	0.14; 0.65	0.23*	− 0.02; 0.48
Smoke (yes vs no)	− 0.48***	− 0.80; − 0.16	− 0.43***	− 0.75; − 0.11
MMT model (public facility vs private facility)	0.29**	0.04; 0.54		
Had received MMT treatment at other MMT facility (yes vs no)	0.20	− 0.05; 0.46		
MMT service satisfaction	− 0.13**	− 0.27; − 0.00		

***p < 0.01, **p < 0.05, *p < 0.1

## Discussion

This is one of the first studies that has examined the preferences of MMT patients for public and private models. This study found the same percentage of patients receiving MMT treatment in a public model and private model. Evaluation criteria of MMT models could be summarized in two dimensions, namely “Availability and convenience of service” and “Competencies of clinic and health professionals.” Self-employed patients were more likely to consider these two dimensions when choosing MMT models. MMT patients in the public model were more likely to choose attitudes of health workers as the reason to use current MMT facility and reported higher service satisfaction than those in the private model.

When choosing an MMT facility, most patients would consider whether this facility is capable of providing comprehensive care, has convenient opening hours, and can treat other diseases. The MMT model, which integrates different components of health care services into a single site, will help address the unmet needs of patients for medical services and improve health outcome [17, 18], as well as reduce patient’s health care expenditure [19–21]. Thus, a convenient and integrative MMT model will be much more popular among MMT patient. We also found that very few patients considered privacy when choosing MMT facilities. In Asian culture, drug users are likely to experience isolation and rejection by society [22, 23]. In this study, privacy was not an important factor for drug users who are seeking MMT services. This may be because drug use-related stigma in the community might have been improved in Vietnam [24, 25].

In this study, most of the patients were self-employed. Patients who were self-employed were more likely to choose “Availability and convenience of service” and “Competencies of clinic and health professionals.” Similar to the findings from a previous study [26], our patients had a high employment rate, but most of them had unstable jobs. It has been reported that over 80% of patients on methadone in Vietnam are not able to participate in stable and long-time employment [5, 27], and they were mainly employed in low-skill jobs, such as being a freelancer or self-employed. Self-employed individual are more conscious of their finances, and hence are more focused on the quality of the service provided, as they have had to bear the cost of the services.

We found patients in a public MMT facility had higher service satisfaction. A previous study reported that older age, higher education, having any problem in self-care, and anxiety/depression were negatively associated with patient’s satisfaction [28]. In our study, there were no significant differences in sociodemographic information between private facility patients and public facility patients, and patient’s age in public facility (median age 41) was slightly higher than that in private facility (median age 38). Older patients tended to have higher expectations and requirement for the service [28]. However, our results showed that patients in a public MMT facility still had higher service satisfaction than in a private MMT facility, though they were older. One of the possible explanations is that patients perceive health workers’ attitudes to be more positive in a public MMT facility than in a private MMT facility. This is consistent with what we found in this study that more public facility patients would choose health workers’ attitudes as a reason to use the current MMT facility. Overall, patients are satisfied with MMT services in Vietnam, and it has been shown that the quality of MMT services in Vietnam has been improved over the past few years [4, 29].

Our findings in this study have implications for policymakers and healthcare providers to maximize the efficiency of MMT treatment in the context of limited resources setting in Vietnam. Firstly, the availability and convenience of service and competencies of clinic and health professionals need to be considered when implementing different MMT service models. Integrating MMT with other health services is important. Secondly, private MMT model needs to improve their level of service satisfaction. More research needs to be conducted to identify patients’ expectations and experiences in MMT. This could help enhance health care quality [30].

Several limitations need to be mentioned. First, convenience sampling was used in this study. This may limit the generalization of the study. Second, our data were based on participants’ self-reports. Recall bias may affect the results. Finally, this is a cross-sectional study. The causal relations between MMT models and related factors could not be ascertained.

## Conclusion

In conclusion, this study highlights the preferences of MMT patients for public and private models. Evaluation criteria about MMT models could be summarized in two dimensions, namely “Availability and convenience of service” and “Competencies of clinic and health professionals.” Compared to a public MMT model, a private MMT model may need to enhance their services to improve patient satisfaction.

## Data Availability

The datasets used and analyzed during the current study are available from the corresponding author on reasonable request.

## Abbreviations

EFA: Exploratory factor analysis
HIV: Human immunodeficiency virus
HIV/AIDS: Human immunodeficiency virus and acquired immune deficiency syndrome
MMT: Methadone maintenance treatment
WHO: World Health Organization
